# 90‐yttrium‐ibritumomab tiuxetan consolidation of fludarabine, mitoxantrone, rituximab in intermediate/high‐risk follicular lymphoma: updated long‐term results after a median follow‐up of 7 years

**DOI:** 10.1002/cam4.684

**Published:** 2016-03-14

**Authors:** Beatrice Casadei, Cinzia Pellegrini, Alessandro Pulsoni, Giorgia Annechini, Amalia De Renzo, Vittorio Stefoni, Alessandro Broccoli, Letizia Gandolfi, Federica Quirini, Lorenzo Tonialini, Alice Morigi, Lisa Argnani, Pier Luigi Zinzani

**Affiliations:** ^1^ Institute of Hematology “L. e A. Seràgnoli”University of BolognaBolognaItaly; ^2^ Department of Cellular Biotechnologies and Hematology“La Sapienza” UniversityRomaItaly; ^3^ Division of Hematology“Federico II” UniversityNapoliItaly

**Keywords:** Advanced, follicular lymphoma, long‐term results, radioimmunotherapy

## Abstract

Radioimmunotherapy (RIT) after an induction phase with conventional chemoimmunotherapy became an attractive strategy of consolidation for patients with advanced follicular lymphoma: in particular, in many studies RIT was represented by yttrium‐90‐ibritumomab tiuxetan (^90^Y‐IT). Independently by the different front‐line treatment, updates on the long‐term follow‐up of these studies are needed because the disease course of follicular lymphoma is characterised by multiple relapses and progressively shorter durations of response. We report updated long‐term efficacy and toxicity results of a multicenter phase II study on sequential treatment with four cycles of fludarabine, mitoxantrone, and rituximab followed by ^90^Y‐IT as front‐line therapy for untreated patients with intermediate/high‐risk follicular lymphoma. With a median follow‐up of 84 months, only 19/49 (38.8%) complete response patients relapsed, yielding an estimated long‐term disease‐free survival of 62.6%. The 7‐year overall survival was 72.7%. Four (7.3%) second acute myeloid leukemia occurred, with a median time following RIT of 42 months. A relevant patients' responsiveness to subsequent therapies occurred: approximately 65% of relapsed patients obtained a good clinical response after the second‐line treatment. These data represented the first evidence of a real role even in the long period of 90Y‐IT after a fludarabine‐containing regimen plus rituximab in the treatment of high‐risk follicular lymphoma.

## Introduction

Follicular lymphoma (FL) is the second most common non‐Hodgkin lymphoma (NHL) and makes up 22% of NHL cases according to the NHL Lymphoma Classification Project 1. FL is characterised by multiple relapses and progressively shorter response durations as the number of therapies increases. Despite the development of numerous treatment strategies to reduce the risk of progression, optimal therapeutic strategies for patients with FL remain undefined. Multiple studies have demonstrated a clinical benefit in treating patients with FL with the radioimmunotherapy (RIT) regimen yttrium‐90 ibritumomab tiuxetan (^90^Y‐IT) 2, 3, 4, including the phase III First‐Line Indolent Trial (FIT): FIT was a prospective, randomised trial that compared observation versus consolidation therapy with ^90^Y‐IT in patients with previously untreated advanced FL who achieved partial response (PR) and complete response (CR) to first‐line induction therapy 5. ^90^Y‐IT was highly effective with no unexpected toxicities, producing a statistically longer time to progression in both PR and CR patients. Subsequently, several phase II studies including first‐line chemotherapy with/without rituximab reported high rate of conversion from PR to CR with significant improvements in progression‐free survival (PFS) 6, 7, 8, 9. Moving from these results, and on the basis of the innovative approach that combines induction chemoimmunotherapy and subsequent consolidation with ^90^Y‐IT with the shortening of chemotherapy duration (to decrease the potential long‐term side effects of antiblastic drugs), some reports have demonstrated interesting results 10, 11, 12, 13. One of these represented our experience: a prospective, multicenter, nonrandomised phase II trial of short fludarabine, mitoxantrone, and rituximab (FMR) induction (only four cycles instead of the conventional six) followed by ^90^Y‐IT in untreated, intermediated/high‐risk FL patients was conducted to evaluate efficacy and safety of this combined regimen 13.Long‐term evaluations of both efficacy and safety now are needed to best integrate this option into current FL treatment algorithms: we now report updated results of the study after a median follow‐up of 7 years.

## Patients and Methods

### Patients

A total of 55 patients with intermediate/high‐risk FL were enrolled between December 2006 and November 2008, at three Italian institutions. The criteria for patient eligibility were previously reported 13. Patients' characteristics are summarised in Table 1. Briefly, patients had to be previously untreated and to present with clear indication to therapy; they had to have a WHO performance status of two or less and Follicular Lymphoma International Prognostic Index (FLIPI) 14 score ≥2. All diagnostic biopsies were reviewed in accordance with the WHO classification 15; the Ki‐67 value ranged from 5% to 20%.

**Table 1:** Patients' characteristics (*n* = 55)

Median age (range) (years)	56 (26–84)
Sex	
Male	25
Female	30
Bulky disease (≥10 cm)	
Yes	11
No	44
Extranodal involvement	
Yes	8
No	47
Stage (Ann Arbor)	
III	20
IV	35
Hemoglobin concentration (g/L)	
<120	22
≥120	33
Number of nodal areas	
>4	20
≤4	35
Increased LDH concentration	
Yes	26
No	29
B symptoms	
Yes	18
No	37
Bone marrow involvement	
Yes	30
No	25
FLIPI (score)	
Intermediate risk (2)	36
High risk (≥3)	19

LDH, lactate dehydrogenase; B symptoms, fever, weight loss, night sweats, pruritus *sine materia*; FLIPI, Follicular Lymphoma International Prognostic Index.

All patients were notified of the investigational nature of this study and signed a written informed consent approved in accordance with institutional guidelines, including the Declaration of Helsinki; the study was approved by each institutional review board.

### Study design

Treatment schedule was the following: 25 mg/m^2^ standard i.v. fludarabine on day 1–3, 10 mg/m^2^ i.v. mitoxantrone on day 2, and rituximab (375 mg/m^2^ on day 1) administered on a 28‐day schedule for four cycles. After the completion of the fourth cycle of FMR regimen, patients were restaged by computed tomography (CT) scan, positron emission tomography (PET) scan, and bone marrow biopsy. Patients were eligible for ^90^Y‐IT if at least in PR after induction, with normal platelet and granulocyte counts and a bone marrow infiltration ≤25%.

After the restaging procedures, eligible patients received one course of ^90^Y‐IT within 12 weeks. It consisted of an infusion of rituximab at 250 mg/m^2^ on day 1 and a subsequent infusion at the same dose on day 8; this second infusion of rituximab was then followed by a weight‐based dose of ^90^Y‐IT, administered at the dose of 11.1 MBq/kg (0.3 mCi/kg) for patients with pretreatment platelet counts ranging from 100 × 10^9^/L to 149 × 10^9^/L, and (0.4 mCi/kg) for those with counts of 150 × 10^9^/L or higher (^90^Y‐IT was routinely administered on an outpatient basis).

### Follow‐up procedures

Strict follow‐up procedures were shared among the three hospitals at the time of the start of the study. The original protocol was amended to extend follow‐up until 8 years from end of treatment. Disease status was evaluated again 3 months after RIT through physical examination, bone marrow biopsy (if still positive after induction chemotherapy), CT and PET scan; other clinically relevant information, such as the development of febrile neutropenia, the use of antibiotics or G‐CSF or blood transfusion during cytopenia, and the presence of any extrahematologic toxicity, were recorded. Patients' follow‐up assessment included: blood count and physical examination every 3–4 months for the first 2 years and then every 6 months for the following 3 years, CT scan every 6 months for the first 2 years, followed by physical examination and annual imaging studies with CT or PET/CT up to 8 years from treatment completion. The determination of tumor response was based on the revised response criteria for malignant lymphoma 16.

### Endpoints

Endpoints of long‐term efficacy were PFS, disease‐free survival (DFS) and overall survival (OS) (16). Endpoints of late toxicity were the incidence and the time of occurrence of second malignancy both hematologic and nonhematologic. The crude incidence of second malignancies was calculated as the proportion of patients diagnosed with second malignancy in the entire study population.

Analyses were conducted on an intention to treat basis.

## Results

**Figure 1:**
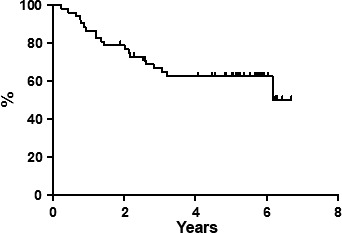
Progression‐free survival.

**Figure 2:**
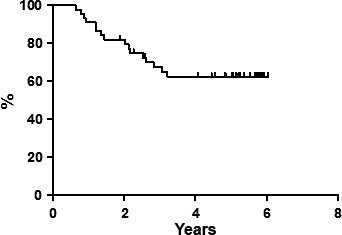
Disease‐free survival.

**Figure 3:**
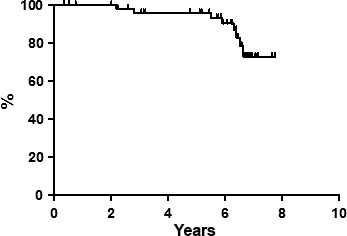
Overall survival.

At the end of the entire treatment regimen (four cycles of FMR regimen plus ^90^Y‐IT), 49 out of 55 (89.1%) patients obtained CR. Median follow‐up at the time of the last analysis was 84 months (range, 10–144 months). All patients completed the scheduled follow‐up period. Seven‐year PFS was estimated to be 50.1% (Fig. 1), and DFS was 62.6% (Fig. 2); 7‐year OS was estimated to be 72.7% (Fig. 3), with eight deaths. There were not distinguishing characteristics between responder and no‐responder patients; no statistically significant difference was observed between patients with FLIPI 2 or higher, either in the type of response or in the long‐term outcomes.

Regarding the late hematological side effects, second malignancies occurred in four (7.3%) patients: all of them reported a secondary acute myeloid leukemia (AML), developed after 36, 38, 45, and 52 months following RIT, respectively (all these four patients died). No late solid tumor malignancies occurred.

Currently, 30 patients (61.2%) are in continuous CR after >5 years of follow‐up. Nineteen patients relapsed during the follow‐up period and four patients died due to lymphoma progression. All the 19 relapsed patients underwent second‐line treatment and received rituximab as part of their second‐line therapy mainly combined with chemotherapy regimens, including: bendamustine (nine cases); doxorubicin, cyclophosphamide, vincristine, and prednisolone (CHOP) (six cases); cyclophosphamide, vincristine, and prednisolone (CVP) (two cases); or ifosfamide, epirubicin, and etoposide (IEV) (two cases). Subsequently, high‐dose therapy with rescue of peripheral stem cells was performed in five patients. No failed attempts at cell harvesting were observed. The overall response rate to second‐line therapy was 63.2% (12/19 patients).

## Discussion

One of the most important challenges for new FL therapeutic options is that they should increase survival without increasing toxicity. The majority of FL patients have advanced disease at diagnosis; in this subset, the advent of rituximab as part of the front‐line approach has changed the conception of FL treatment. There is no international consensus on the optimal front‐line chemotherapy regimen for patients with advanced FL, and currently, the most widely used front‐line treatment for patients with FL is rituximab in combination with chemotherapy 17, 18, 19, 20.

On the other side, radioimmunotherapy after the induction phase with conventional chemoimmunotherapy became an attractive strategy of consolidation: in particular, in many studies, radioimmunotherapy was represented by ^90^Y‐IT 5, 10, 11, 12, 13.

Independently by the different front‐line treatment, it should be very important to have an update on the long‐term follow‐up of these studies because the disease course of FL is characterised by multiple relapses and progressively shorter durations of response with a median survival of approximately 10 years 21.

For this reason, our report updates long‐term efficacy and toxicity results of a sequential treatment with four cycles of FMR followed by ^90^Y‐IT as front‐line therapy for untreated patients with intermediate/high‐risk FL.

With the potential benefit of reducing induction cycles from 6 to 4 achieving the same results 6 and with the chance of reducing the cumulative toxicity of the two additional cycles of conventional chemotherapy, this therapeutic regimen confirms its real role even in the long‐term period. Similar updates are very rare 22.

With a median follow‐up of 84 months, only 19/49 (38.8%) CR patients relapsed, yielding an estimated long‐term DFS of 62.6%; particularly, the last relapse in terms of timing was observed within 36 months showing a plateau of DFS curve after this point. Furthermore, it is important to remember that patients were intermediate or high‐risk according to FLIPI score.

There is a lively debate in literature regarding the issue of safety of this regimen. We shortened the course of fludarabine from 6 to 4 cycles and this could be the reason of generally mild and transient toxic effects during and immediately after treatment. No infections occurred 13. The AML incidence with conventional dose chemotherapy or radiotherapy ranges from 0% to 12% in literature and numerous fundamental questions remain unanswered with regard to the actual (as opposed to the actuarial) incidence of treatment‐related AML (tAML), the etiology and the definition of the disease, and the additional patient or treatment‐related risk factors 23. tAML are clinically and cytogenetically distinct from de novo cases: a potential bias of our report is that we did not distinguish between de novo and treatment‐related AML cases. We chose to report crude incidence because we were not able to consider all the competing risks and due to the fact that there is a degree of ambiguity with regard to the statistical methodology used in the literature; several results are often not comparable. However, the crude incidence of t‐AML has been reported to range from 0% to 12% with conventional dose chemotherapy or radiotherapy 24 and the present 7% is within this range. Furthermore, in our previous reports on ^90^Y‐IT no AML occurred so far 7, 25.

So far it is not clear if fludarabine combined with ^90^Y‐IT could really increase the number of leukemias. It is possible that a fludarabine‐free chemotherapy could further reduce the number of leukemias and safety concerns remains a matter of debate.

A relevant patients' responsiveness to subsequent therapies was registered; approximately 65% of relapsed patients obtained a good clinical response after the second‐line treatment. In this subset, the consolidation therapy with ^90^Y‐IT did not appear to rule out any second‐line treatment approach, including harvesting an autologous stem cell graft and subsequent autologous stem cell transplantation (as noted previously also in a specific report) 26.

In conclusion, in this 7‐year update report our data confirmed an impressive DFS higher than those reported for the other chemoimmunotherapies also in long‐term follow‐up although patients were intermediate/high‐risk FLIPI and underwent induction phase with a short course chemoimmunotherapy.

## Conflict of Interest

Authors have nothing to disclose.
